# Habitual physical activity levels do not predict leg strength and power in healthy, active older adults

**DOI:** 10.1371/journal.pone.0200089

**Published:** 2018-07-02

**Authors:** Oliver J. Perkin, Polly M. McGuigan, Dylan Thompson, Keith A. Stokes

**Affiliations:** 1 Department for Health, University of Bath, Claverton Down, Bath, United Kingdom; 2 Arthritis Research UK, Centre for Sport, Exercise and Osteoarthritis, Bath, United Kingdom; McGill University, CANADA

## Abstract

Physical activity is considered crucial in attenuating losses in strength and power associated with ageing. However, in well-functioning, active older adults the relationship between habitual physical activity and muscle function is surprisingly unclear. Leg press velocity, force, and power, were compared between 50 older and 30 younger healthy individuals, and associations with habitual physical activity explored. An incremental power test was performed on a pneumatic leg press, with theoretical maximum velocity, force, and power calculated. Vastus lateralis muscle thickness was measured by ultrasound, and participants wore a combined accelerometer and heart rate monitor for 6-days of free-living. Older individuals produced lower absolute maximum velocity, force, and power, than younger individuals. When accounting for smaller muscle size, older individual’s maximum force and power remained markedly lower. Both groups were active, however using age specific thresholds for classifying physical activity, the older individuals engaged in twice the amount of moderate-to-vigorous physical activity in comparison to the younger individuals. There were no associations between any characteristics of muscle function and physical activity. These data support that the ability to generate force and power deteriorates with age, however habitual physical activity levels do not explain inter-individual differences in muscle function in active older individuals.

## Introduction

Ageing is associated with decreasing skeletal muscle mass at a rate of around 0.5–1% per year after the age of 50 years, with concomitant loss of muscle force generating capability [1]. This loss of muscle mass (sarcopenia) and strength (dynapenia), particularly in the lower limb, are regarded as central causes of functional decline and subsequent loss of independence in older adults [2, 3].

It is evident that age related muscle loss is associated with an uncoupling of the relationship between muscle size and force generating capacity that would normally be observed in younger healthy individuals [4, 5]. Loss of isokinetic knee extensor torque occurs at 2–5 times the rate that would be expected based on muscle mass loss [6]. Furthermore, maximum contraction speed of muscle is also observed to decrease with advancing age [7]. As such, the rate of force transfer from muscle (i.e., power) is lost to a greater extent than maximal force producing capacity alone [8, 9]. In day-to-day living, voluntary movements requiring relatively high force production are likely to require rapid execution, such as in trip recovery, thus in an ageing population muscle power may be a more useful measure than isometric or isokinetic strength. Indeed, power is considered a better predictor of functional status than muscle mass or strength alone, and is more sensitive to age-related physiological changes such as impaired neuromuscular activation and changes in muscle architecture [10, 11]. Accurate and safe measurement of peak power in older adults is challenging due to safety and technique related issues, however use of pneumatic resistance exercise machines instrumented to record kinetic data has emerged as an accurate, valid and reliable tool to assess lower limb muscle power [12].

Maintaining an adequate level of physical activity is regarded as crucial for preserving muscle function into later years of life [13]. At the single fibre level contractile properties of muscle of healthy men aged over 70 years are protected to some degree by physical activity (walking at least two hours a day) compared to being sedentary [14]. However, at the whole body level the relationship between habitual physical activity and muscle function during ageing is surprisingly less clear. In mobility-limited older men, leg press strength was weakly correlated with physical activity level counts from a waist mounted accelerometer, but leg press power was not [15]. Similarly, no association was found between changes in isokinetic knee extensor strength and physical activity over 10 years in a wide range of physically functioning older men and women (aged ~70 years at follow-up) [16]. The authors suggested that this cohort may have been above a threshold at which the habitual physical activity levels assessed by questionnaire would have a direct effect on muscle strength. However, in community dwelling women aged 74 (6) years, daily moderate intensity physical activity levels, assessed by questionnaire, were associated with lower limb power independent of lower limb muscle mass or resistance training [17]. Overall, current understanding of the relationship between habitual physical activity levels and lower limb muscle power in active, healthy older individuals is incomplete and often limited by the self-report techniques that have been used to date.

The aim of the present study was to compare characteristics of muscle function (maximal velocity, force, and power) during bilateral leg press exercise between healthy older and younger individuals and to explore relationships between muscle function characteristics and habitual physical activity using multidimensional physical activity monitoring.

## Materials and methods

### Participants

Healthy, older (65–80 years) and younger (20–35 years) men and women were recruited for the study through local newspaper advertisement and social media. Potential participants were fully informed as to the study design and methodology with written information, and a telephone interview was conducted by a member of the research team to ascertain initial eligibility. Individuals who were non-smokers, BMI <30 kg/m^2^, not regularly taking anti-inflammatory medication, and had no contraindications to exercise or recent history of musculoskeletal injury were invited to the laboratory to undertake the Short Physical Performance Battery (SPPB) [18]. Individuals scoring 8 or above, and not scoring zero on any particular SPPB test, were included in the study. All participants provided written informed consent. The protocol was approved by the University of Bath Research Ethics Approval Committee for Heath. Participant characteristics are presented in Table 1.

**Table 1:** Participant characteristics.

	Older (*n* = 50; ♀ = 25)	Younger (*n* = 30; ♀ = 15)
**Age (years)**	70 (4)**	25 (4) 26 (4)
**Body Mass (kg)**	69.3 (12.4)	68.5 (10.8) 61.6 (5.2)
**BMI (kg/m^2^)**	24.3 (3.4)*	22.6 (2.8) 21.8 (1.9)
**VL muscle thickness (mm)**	18 (4)**	22 (4) 20.6 (2.2)
**SPPB Score (/12)**	11 (1)	11 (1) 11 (1)

Data presented as mean (standard deviation). BMI = Body mass index; VL = vastus lateralis. SPPB = Short Physical Performance Battery.

* / ** denotes significant differences between age groups (*P < 0.05, **P < 0.01).

### Procedures

Participants visited the laboratory on two occasions separated by at least seven days. Participants were requested to refrain from exercise in the 24 hours prior to testing, and to continue with their normal dietary habits. During the first visit, measurements of vastus lateralis (VL) muscle thickness were taken using ultrasound. Transverse images were taken at 50% of thigh length from the left leg using a 128 element linear array transducer (LV7.5/60/96Z, TELEMED, Lithuania) operating in B-mode at 8.0 MHz. Three images were taken from each participant. Muscle thickness was measured adjacent to the muscle fascia at the thickest part of muscle belly between the inside edges of the muscle fascia in each of the images and a mean calculated for each participant. All images were processed by the same researcher (OJP) using ImageJ software (1.44p, Wayne Rasband, National Institutes of Health, USA).

### Force and power

A Keiser A420 seated pneumatic leg press dynamometer (Keiser®, Fresno, CA) was used to measure lower limb force and power production characteristics using the manufacturer’s software. The seat position was set such that pre-repetition knee angle was approximately 90° and the participant was comfortable. The same seat position was used in both trials. During the preliminary trial, participants performed a one-repetition maximum (1-RM) test in which discrete repetitions to failure were attempted at participant-selected increments in resistance. Repetition velocity and rest periods between repetitions were self-selected, with participants instructed to aim to reach their 1-RM within 20 repetitions. Ten minutes of seated rest were then observed, followed by familiarisation with an incremental power test in which approximately 10 discrete leg press repetitions were performed at maximum voluntary movement velocity for each repetition. Resistance and duration of rest between efforts increased with each repetition and were calculated by the manufacturer’s software, with resistance of the tenth repetition corresponding with the previously achieved 1-RM. For the main trial the same incremental power test was used with the same set of increasing resistances.

Sampling frequency of linear displacement and pressure within the pneumatic pistons (creating resistance at the foot pedals of the leg press machine) was 400 Hz, with pedal velocity (m/s) and resistance force (N) calculated by the manufacturer’s software. These data were sampled for the duration of the concentric phase of each discrete repetition of the incremental power test, with the first and last 5% of pedal displacement discarded from analysis by the manufacturer’s software. Data were sampled independently from each leg, and the mean velocity, force and power from both legs for each repetition used for subsequent analysis. Leg press resistance was provided by pneumatic pistons, thus resistance increased across the range of motion of each repetition, albeit modestly. As such, peak force of a repetition would be dependent on pedal displacement. Consequently velocity and force at the instant of peak power were recorded for each repetition rather than peak velocity and peak force. Data were subsequently analysed manually using Microsoft Excel to determine theoretical maximum force and velocity.

Bobbert [19] demonstrated that the force-velocity relationship of a leg pressing movement is quasi-linear, therefore a linear regression of velocity and force at peak power for each repetition across the incremental power test was calculated, and maximum theoretical velocity (V_max_) and force (F_max_) were extrapolated. Previous studies of muscle function characteristics *in vivo* have often drawn data from participants performing a series of discrete repetitions of an exercise at maximum contraction velocity against increasing external loads [20–22]. Peak power would then be defined as the greatest power achieved in any of the performed repetitions irrespective of the % of 1-RM at which the repetition was performed [11]. However, this approach may lack resolution with peak power likely occurring at an external load between resistances actually attempted. Therefore interpolated peak power (P_max_) was determined by numerical differentiation of the second-order polynomial equation calculated from the force-power profile [23].

### Physical activity

Participants wore a combined accelerometer and heart monitor (Actiheart™, Cambridge Neurotechnology, UK) mounted on the chest using adhesive electrode pads (3M, UK) continuously for six free-living days to record habitual physical activity in one-minute epochs using the default equations in the manufacturer’s software. Participants were instructed to continue with their normal lifestyle, and to remove the monitor only for water based activity. Daily physical activity level (PAL) was calculated as a ratio of total energy expenditure (TEE) to basal metabolic rate (BMR), with BMR calculated using participant height, weight, age, and sex as described by Schofield [24]. Age specific physical activity thresholds were used to categorise physical activity patterns (sedentary time = ≤1.5 METs for both groups, and for the older group moderate to vigorous activity (MVPA) = ≥3.2 METs, and for the younger group MVPA = ≥4.8 METs) [25].

### Statistical analysis

All data were found to be normally distributed according to Shapiro-Wilk tests. There was no effect of sex on the differences between age groups, thus data for both sexes were pooled for each age group. Differences in anthropometric data and muscle function characteristics, PAL, and minutes per day spent within sedentary and MVPA thresholds of physical activity between age groups were assessed with independent samples *t*-test. Hedge’s *g* effect sizes were calculated for between age group differences in muscle function characteristics, PAL, and minutes per day spent within given thresholds of physical activity on account of the unequal group sizes. Pearson’s correlation was used to assess relationships of muscle function (V_max_, F_max_ and P_max)_ with PAL and with minutes of sedentary time and MVPA per day. All statistical analysis was conducted using SPSS v22.0 (SPSS Ins., Chicago, IL). Data are presented as mean (standard deviation) with statistical significance accepted at *P* < 0.05.

## Results

### Muscle function characteristics

**Fig 1:**
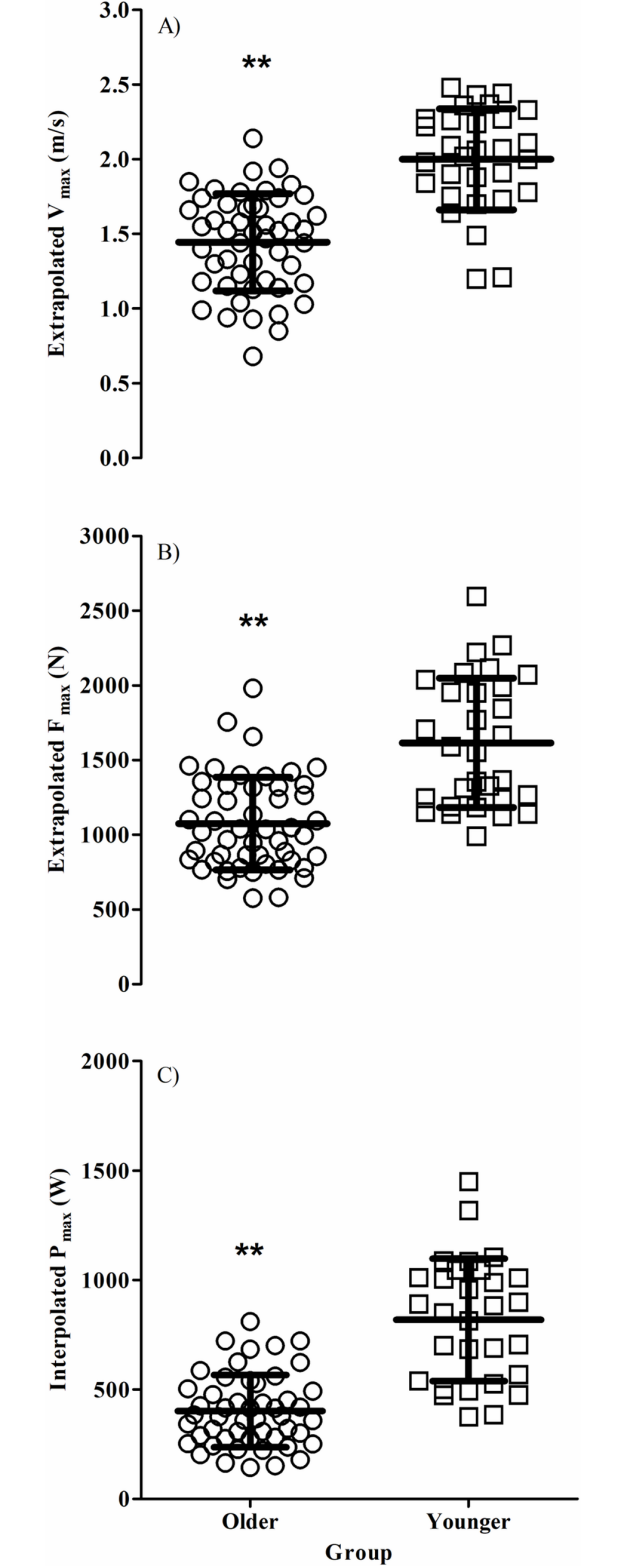
Velocity, force, and power is significantly lower in healthy older individuals than younger individuals. Mean ± SD extrapolated peak A) veolcity (V_max_), B) force (F_max_), and C) interpolated peak power (P_max_) for 65–80 vs 20–35 yr olds. The older individuals are represented by circles and the younger individuals by squares. **denotes significant difference between age groups (*P <* 0.01).

**Fig 2:**
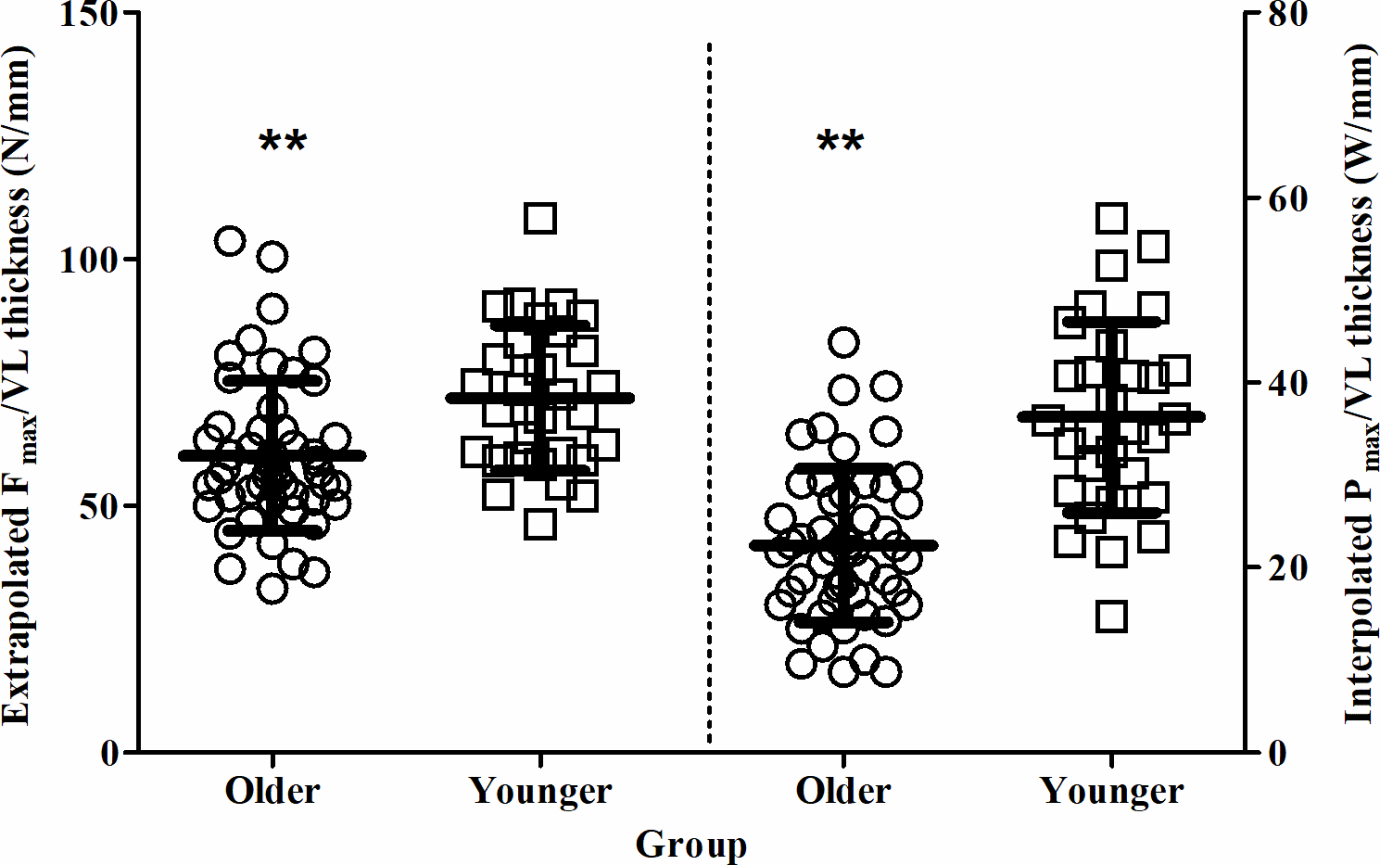
Relative force and power are significantly lower in healthy older individuals than younger individuals. Mean ± SD extrapolated peak force (F_max_:VL) and interpolated peak power (P_max_:VL) relative to vastus lateralis muscle thickness for 65–80 vs 20–35 yr olds. The older individuals are represented by circles and the younger individuals by squares. **denotes significant difference between age groups (*P* < 0.01).

As shown in Fig 1, V_max_ was significantly lower in older individuals compared to younger individuals (1.44 (0.33) m/s vs. 2.00 (0.34) m/s, *P* < 0.01, *g* = 1.09), as were F_max_ (1074 (310) N vs. 1615 (433) N, *P* < 0.01, *g* = 0.98) and P_max_ (402 (165) W vs. 819 (180) W, *P* < 0.01, *g* = 1.59). When F_max_ and P_max_ were considered relative to VL muscle thickness, F_max_:VL was significantly lower in older individuals compared to younger individuals (60.1 (15.2) N/mm vs. 71.8 (14.7) N/mm, *P* < 0.01, *g* = 0.51), as was P_max_:VL (22.4 (8.3) W/mm vs. 36.2 (10.3) W/mm, *P* < 0.01, *g* = 0.93) (see Fig 2).

### Physical activity

Physical activity data for two female participants in the older group could not be obtained due to technical issues, thus participant characteristics for the older group included in the physical activity analyses were: Age 69 (4) years; body weight 69.7 (12.5) kg; BMI 24.4 (3.4) kg/m^2^; VL thickness 18.2 (4.0) mm; and SPPB score 11 (1) /12.

Physical activity level (PAL) was significantly lower in older compared to younger individuals (1.59 (0.17) vs. 1.74 (0.23), *P* < 0.01, *g* = 0.77). Using age specific thresholds for classifying physical activity based on METs, minutes per day spent being sedentary were not different between the older and younger groups (1058 (112) min/d vs. 1017 (128) min/d, *P* = 0.14, *g* = 0.35). Older individuals spent more time engaging in MVPA than those in the younger group (103 (49) min/d vs. 49 (29) min/d, *P* < 0.01, *g* = 1.27), when using age specific physical activity thresholds.

**Fig 3:**
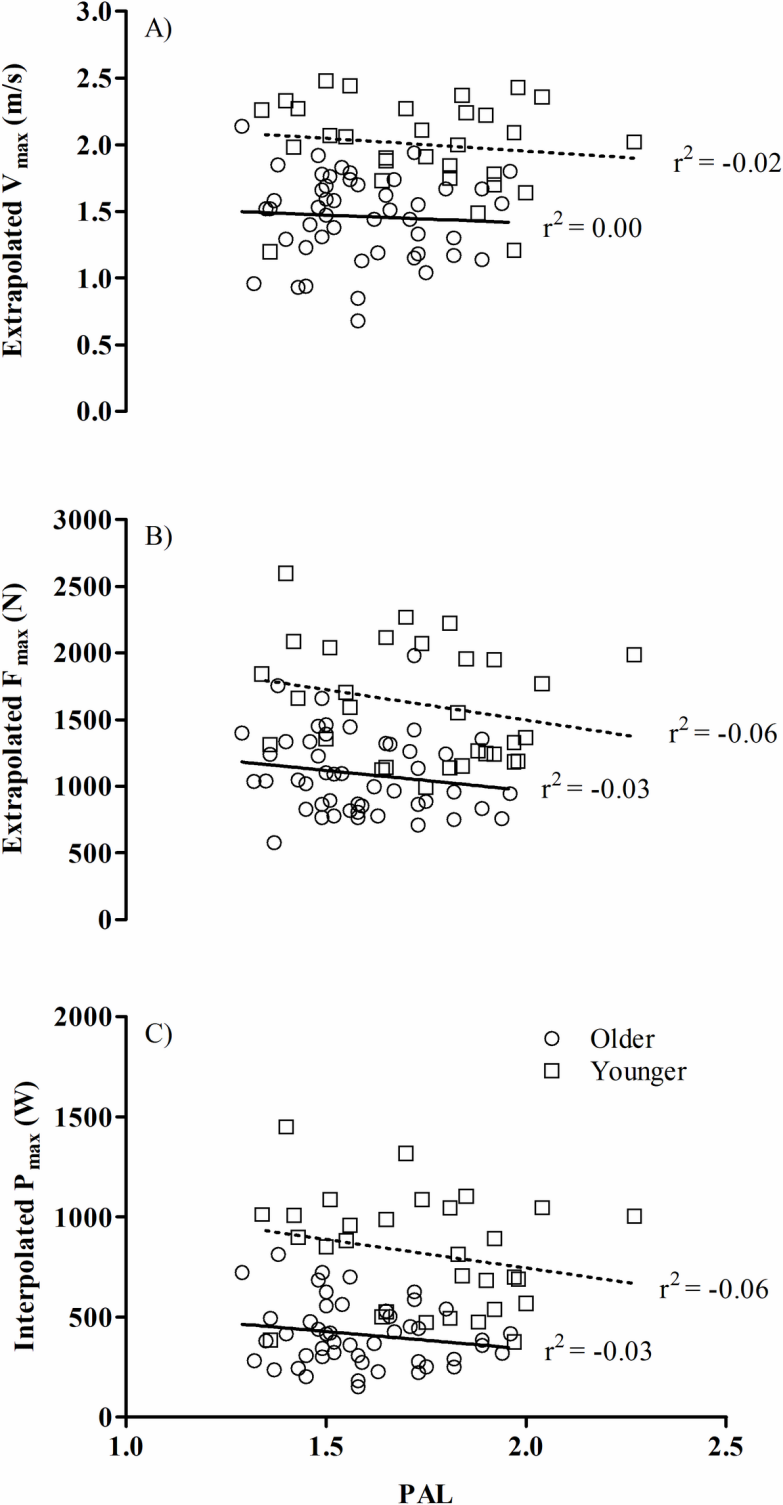
Habitual physical activity level was not associated muscle function in healthy older or younger individuals. Pearson’s correlations between daily physical activity level (PAL) (ratio of total energy expenditure to basal metabolic rate) to A) extrapolated maximum velocity (V_max_), B) extrapolated maximum force (F_max_), and C) interpolated maximum power (P_max_) for 65–80 and 20–35 year olds. The older individuals are represented by circles and the younger individuals by squares. No significant relationships between PAL and muscle function characteristics were identified for either group.

Muscle function (V_max_, F_max_, or P_max_) and PAL were not associated in either the older group or younger group (see Fig 3). Muscle function and minutes per day spent sedentary or engaging in MVPA were not significantly correlated in either group.

## Discussion

The main finding was that maximum movement velocity and force at peak power during leg pressing were lower in older individuals compared to younger individuals, resulting in markedly lower peak power. Older individuals had lower vastus lateralis muscle thickness, but force and power producing capability were also significantly lower per unit of muscle thickness in older compared to younger individuals. Overall physical activity level (PAL) was lower in the older group than the younger group (1.59 (0.17) vs. 1.74 (0.23) respectively), however the older group undertook more daily MVPA than the younger group when applying age-specific cut-offs. Despite the older group being reasonably physically active, neither PAL nor MVPA were associated with muscle function in either group.

It has long been recognised that force producing capabilities of muscle decline with age, and that muscle power is lost at a faster rate than strength [26]. The present data support this; whilst F_max_ was 33% lower in the older compared to the younger group, P_max_ was 51% lower. Vastus lateralis muscle thickness was 19% lower in the older compared to the younger group and therefore lower force and power production would be expected [4]. However, using force or power per unit of VL muscle thickness as an approximation of specific force, F_max_ was still lower by 16% and P_max_ by 38% in the older group. Potential causes of reduced force and power producing capabilities relative to muscle size with ageing include decline in neuromuscular activation [9], architectural changes such as changes in tendon stiffness [27] and fat infiltration [6], and possibly deterioration of specific force at the single fibre level [14]. Irrespective of the mechanism, deteriorating muscle power in older age represents a cause for concern. Analysis of factors related to functional status in older women identified leg muscle power as the strongest univariate correlate of functional status, above maximum oxygen uptake capacity, muscle strength, physical activity levels, and even age [20]. Furthermore, leg muscle strength has been demonstrated to predict mortality over six years of follow-up in men and women over the age of 70, despite no association between mortality and muscle mass [28].

The older group in the present study represented a well-functioning (SPPB score of 11 (1)) and reasonably active population (PAL = 1.59 (0.17)) [29]. In ‘absolute’ terms, physical activity accounted for proportionally less of the daily TEE in the older group, however the older group performed more than twice the daily MVPA relative to the young group. Despite this, habitual physical activity levels did not correlate with muscle function, either in absolute terms (F_max_ and P_max_) or when considered per unit of VL muscle thickness (data not shown). Notwithstanding the intuitive appeal of physical activity being positively associated with muscle strength into older age, self-reported physical activity had no association to maximal leg muscle strength in 850 elderly adults, although it was strongly predictive of general motor function [30]. Furthermore, it would seem that even declining physical activity longitudinally assessed over 10-years by questionnaire does not directly explain declines in strength in relatively active older adults [16]. The present findings demonstrate that even accurate and objectively measured PAL or time spent engaging in MVPA are not associated with maximal muscle function capabilities in healthy, active >65 year olds.

It is not clear why habitual physical activity was not predictive of muscle function characteristics. It may be that the specific movements in everyday life that could be important for muscle strength or power do not contribute to overall physical activity energy expenditure. Indeed, whilst the Actiheart™ device is an accurate and reliable measure for assessing habitual ambulatory physical activity [31], these devices cannot capture ‘natural’ resistance/loading exercise such as stair climbing or hill walking with additional load of shopping bags for instance. Moreover, it may be that relatively well-functioning older individuals such as those in the present study may be above a threshold at which habitual physical activity impacts muscle function characteristics directly [16]. In any case it should be emphasised that the present data does not undermine the wealth of evidence for the importance of maintaining a physically active lifestyle into older age [32]. In a more heterogeneous sample of older adults the variability in muscle function may be associated with habitual physical activity level [33]. Recent data examining force-velocity profiles from leg pressing and performance of physical and cognitive function, frailty, and health-related quality of life in older adults adds some support to this notion. For example, in the present study SPPB score ranged from 8–12, whereas in a group of 70–85 year olds with SPPB scores ranging from 4–12, MVPA was positively associated with F_max_ and P_max_ [34]. Indeed, the dramatic impact on muscle function and mass of removing, or even just reducing, daily activity for a short period of time also explify this [35, 36]. The present data do highlight that lower limb muscle function in terms of maximal velocity, force, and power producing capabilities, is impaired in older individuals compared with younger individuals even when remaining active, and focus needs to be placed on recognised strategies to maintain muscle function such as resistance exercise [37, 38]. Moreover, greater strength and power are themselves associated with improved health outcomes in later life, and essentially facilitate that tasks of daily living are in relative terms less strenuous, thus likely delaying onset of frailty [12].

There are limitations to consider in the present findings: for example a measure of all leg extensor muscle volume would have been a more accurate basis for assessing muscle force producing capability relative to size than ultrasound measurement of VL alone [4]. No measures of motor unit recruitment efficacy (for example twitch interpolation) or muscle fibre type characteristics were recorded, which would have provided insight into the mechanism by which force and power producing capability degenerate with ageing [39, 40]. Additionally, whilst outcomes such as muscle function remain relatively stable, with physical activity levels being inherently malleable the Hawthorne effect may have impacted activity patterns, despite participants being instructed to maintain their regular lifestyle [41]. Whether this would have differed between groups cannot be estimated. The Actiheart™ device is not waterproof, thus would have been removed during any water-based activities or exercise, and does not provide data on wearer posture. Information on water-based exercise and better characterisation of sedentary behaviours, i.e. lying or sitting versus standing, could have been achieved with the use of an activity diary to accompany the physical activity monitor. Furthermore, the potential for seasonal variation in activity and indeed the lifetime history of physical activity should be considered in future work [42].

In conclusion, velocity and force producing capability of muscle decreases with age even in active older adults, which translates to a reduction in muscle power production both in absolute terms and relative to muscle size. However, the variability in muscle function characteristics during dynamic leg pressing was not explained by objectively measured habitual physical activity. As such, whilst total physical level is important for many indices of healthy ageing, targeted efforts such as resistance exercise are likely a requirement to maintain muscle function into later life.
